# Visualizing highly cited scientific output of Indian physiotherapists: A bibliometric study

**DOI:** 10.12688/f1000research.22390.2

**Published:** 2020-05-19

**Authors:** Arun Vijay Subbarayalu, Manuelraj Peter, Mohamed Idhris, Sivasankar Prabaharan, Muhil Sakthivel, Vinoth Raman, Palanivel R.M., Ola Ibrahim Ramzi

**Affiliations:** 1 Quality Measurement and Evaluation Department, Deanship of Quality and Academic Accreditation, Imam Abdulrahman Bin Faisal University, Dammam, Saudi Arabia; 2 Library Systems Department, Deanship of Library Affairs, Imam Abdulrahman Bin Faisal University, Dammam, Saudi Arabia; 3 Institutional Repository Department, Deanship of Library Affairs, Imam Abdulrahman Bin Faisal University, Dammam, Saudi Arabia; 4 Measurement & Evaluation Unit, Office of Vice Presidency for Graduate Studies & Scientific Research, Imam Abdulrahman Bin Faisal University, Dammam, Saudi Arabia; 5 College of Public Health & Academic Accreditation Department, Deanship of Quality and Academic Accreditation, Imam Abdulrahman Bin Faisal University, Dammam, Saudi Arabia

**Keywords:** Bibliometric study, India, Physiotherapy, Scientific output

## Abstract

**Background: **Physiotherapy research supports the advancement of evidence-based practice and the development of a highly skilled workforce. This study aims to visualize the highly cited scientific output of Indian physiotherapists from 1999 to 2018.

**Methods: **A descriptive study design was adopted to visualize the highly cited scientific output of Indian physiotherapists using the Web of Science (WoS) database from 1999 to 2018. A search was carried out using the following keywords "((TS=(physiotherapy) OR TS=("physical rehabilitation") OR TS=("physical therapy")) AND AD=(India))Indexes=SCI-EXPANDED, SSCI, A&HCI, CPCI-S, CPCI-SSH, ESCI, CCR-EXPANDED, IC Timespan=1999-2018”. Data collected were analyzed using Incites from WoS and VOSviewer software.

**Results:**  A total of 488 articles were published between 1999 and 2018, with a peak of 103 in 2016 with 2419 citations. A decline in publication count was observed after 2016. The journal *International Journal of Physiotherapy* published the highest number of articles (n=35). Manipal University (n=36) was found to be the most active institution for physiotherapy research in India, as determined by publishing the most articles. Indian physiotherapists published the highest number of research articles in collaboration with US authors (n=24).

**Conclusion:** There is an increasing trend in the scientific output of Indian physiotherapists over the past two decades; however, a decline is observed after 2016. It is recommended that research collaborations across the globe are increased and scientific output should be improved, leading to a higher number of citations. Future research should explore factors influencing the scientific production of Indian physiotherapists and devise appropriate strategies to attain further improvement.

## Introduction

The scientific output of a profession is recognized by the frequency of publications, which are published in peer-reviewed journals and indexed in bibliographic databases ^1– 3^. In physiotherapy, this scientific output is utilized to enhance existing knowledge and develop guidelines for highly effective clinical practice ^4^. Accordingly, the analysis of scientific output allows the definition of baseline indicators in knowledge and clinical practice in physiotherapy ^5, 6^. Various studies investigated the scientific output of physiotherapists across the globe ^6– 14^. Among these studies, several utilized electronic searches ^6, 7, 9– 11, 13, 14^, whereas others were limited to document reviews ^8, 12^. Concerning the Indian context, only two studies have been performed to reveal the research productivity of Indian physiotherapists from 2000 to 2014, which were limited to the Medline database ^10, 11^. Moreover, Li *et al.* (2018) recently stated that Clarivate Analytics’s Web of Science (WoS) is the World’s foremost scientific citation search and analytical platform, which can be used as both a research tool and dataset ^15^. Hence, there is a need for further research that should involve the WoS database to detect high-quality research publications by Indian physiotherapists until 2018. Therefore, this study was planned to conduct a bibliometric study on the scientific output of Indian physiotherapists using WoS during the last two decades (from 1999 to 2018).

## Methods

The descriptive study design was adopted to reveal the scientific output of Indian physiotherapists using an electronic literature search in the WoS database during the period from 1999 to 2018.

### Article selection

The search was conducted on 14th October 2019. The term ‘Indian physiotherapists’ denotes physiotherapy professionals employed in any of the academic or clinical establishments in India in the study period. The search was carried out in WoS using the following keywords “((TS=(physiotherapy) OR TS=(“physical rehabilitation”) OR TS=(“physical therapy”)) AND AD=(India))Indexes=SCI-EXPANDED, SSCI, A&HCI, CPCI-S, CPCI-SSH, ESCI, CCR-EXPANDED, IC Timespan=1999–2018”. The search started from 1999 since this study aimed to retrieve data from the past two decades.

### Article screening

**Figure 1.**
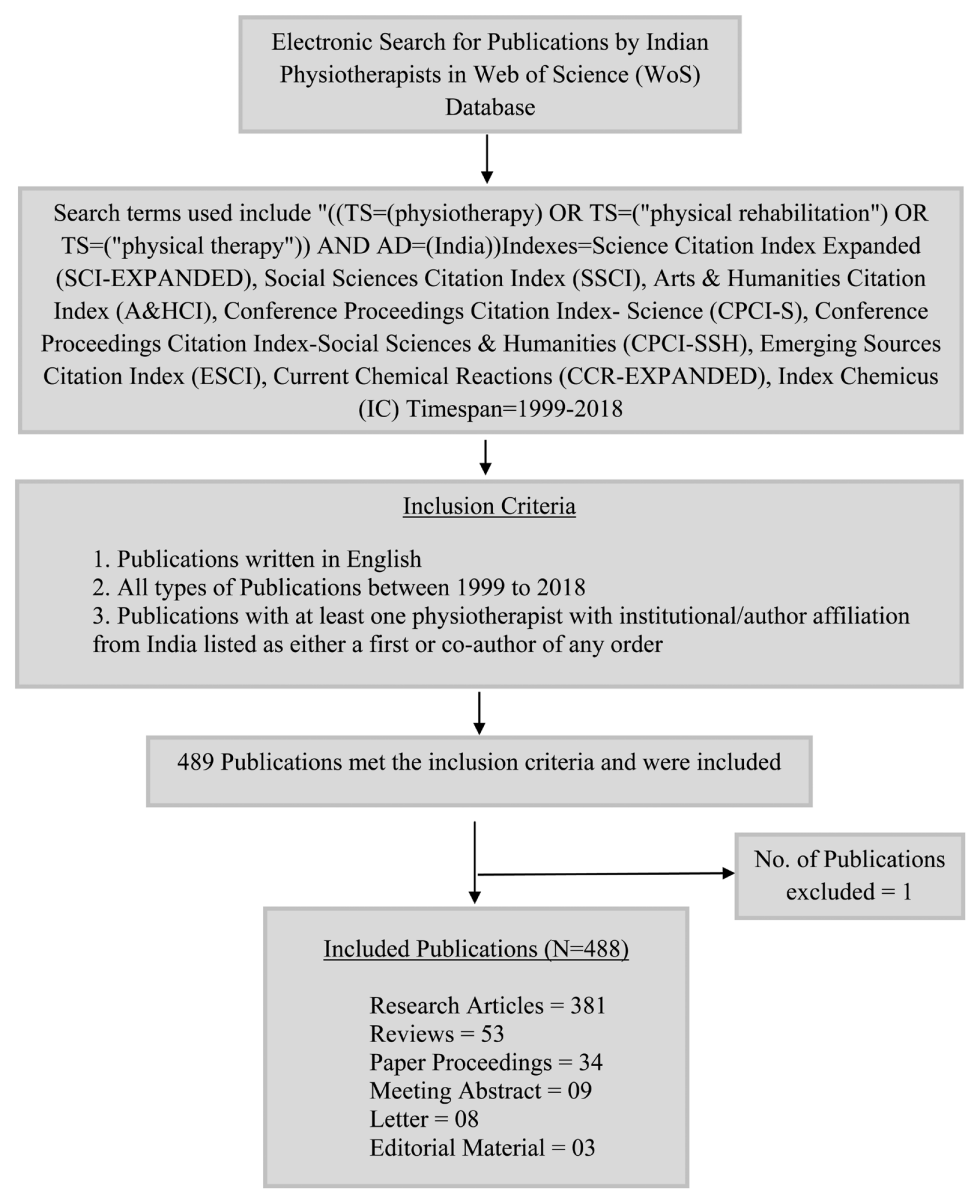
Flowchart showing the process for search methodology.

The search methodology is described in Figure 1. Based on the inclusion criteria, 488 publications were included and proceeded for further analysis.

### Data analysis

**Figure 2.**
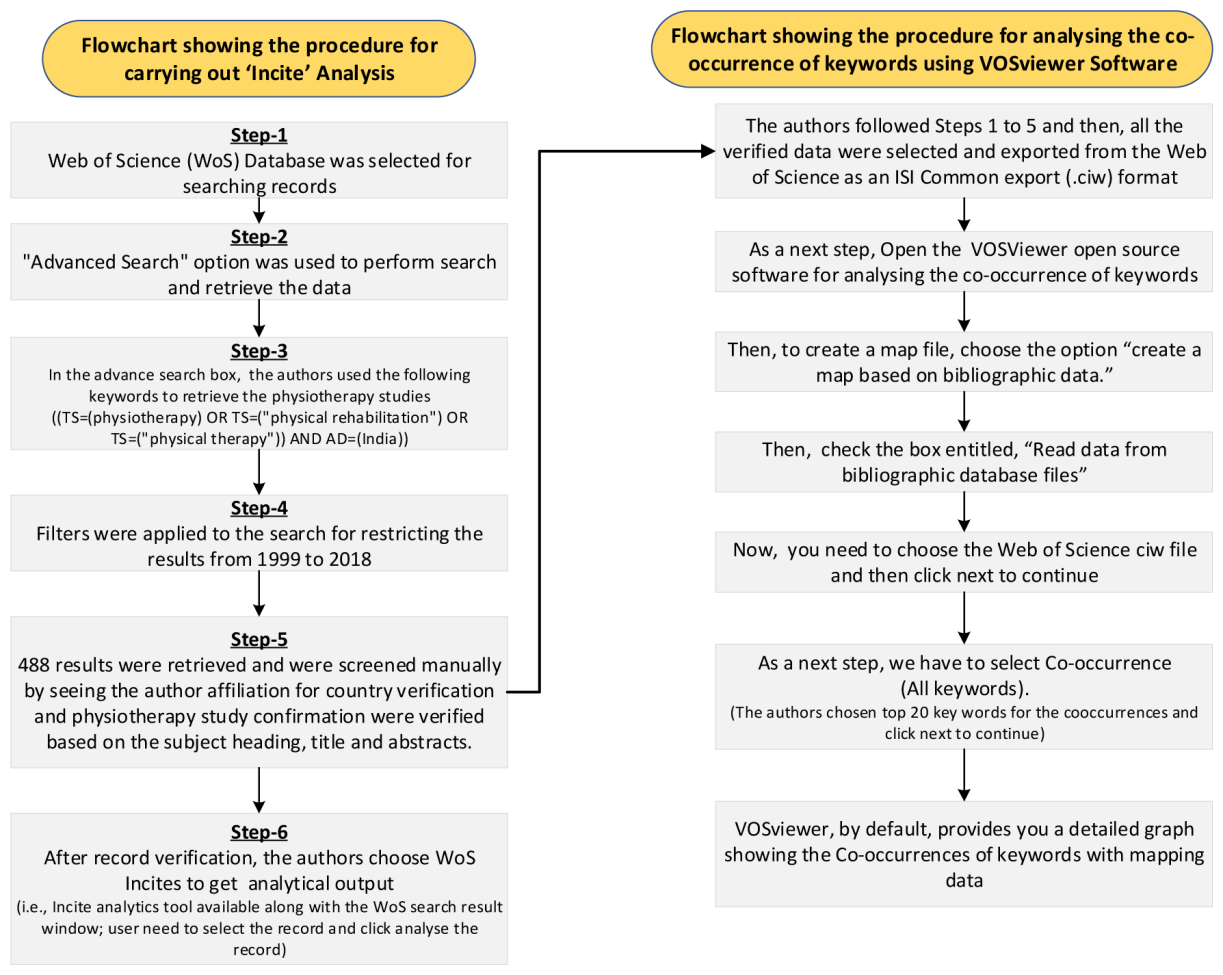
Flowchart showing the procedures to execute the Incites and VOSviewer analysis.

Retrieved articles were analyzed using Incites in WoS and Visualization of Similarities (VOS) viewer 1.6.11. VOS is a new method used for visualizing similarities between objects ^16, 17^. Incites was used to gather information on publication year, authorship ranking, source journal productivity, collaborating institutions, country-wise research collaboration, citations, and collaboration pattern of articles. In addition, the information related to h-index was obtained from the Incites in WoS. Here, the h-index reflects the productivity of authors based on their publication and citation records. It is useful because it discounts the disproportionate weight of highly cited papers or papers that have not yet been cited. The data, which is exported from the WoS database as an ISI common export (.ciw) format, were imported into VOSviewer to explore the co-occurrences of keywords used by the authors in their articles. The flowchart describing the procedures for carrying out both Incites and VOSviewer analysis is depicted in Figure 2.

Since Incites in WoS used in this study is a proprietary software, the researchers could alternatively use a tab-delimited file downloaded from WoS and use it in VOSviewer.

## Results

**Figure 3.**
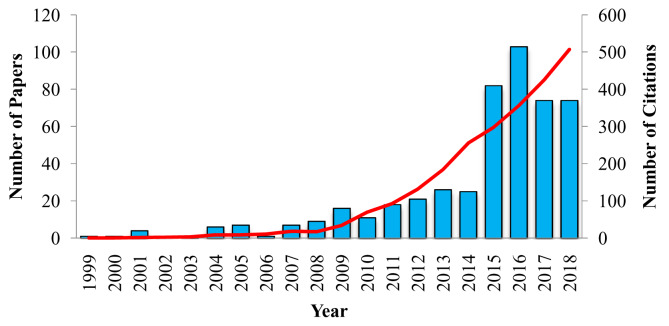
The growth trend for publications and citations by Indian physiotherapists between 1999 and 2018. Data obtained from Web of Science. Includes all articles types.

A total of 488 articles were included in the study; 381 research articles, 53 reviews, 34 proceedings, 9 meeting abstracts, 8 letters, and 3 editorial materials. The chosen period of study was divided into four strata with five years each. The strata were 1999–2003; 2004–2008; 2009–2013; and 2014–2018. In the first two strata, the publication count was observed in a single digit (≤9). In the third strata, this count has reached two digits with a maximum of 26 in 2013. However, in the fourth strata, an abrupt rise in the publication count was observed with a peak of 103 in 2016. Notably, the publication counts after 2016 decreased and rose slightly in 2018, but not to those levels seen in 2016 ( Table 1 and Figure 3). Regarding citation count, there was a gradual rise over the research period, with a total of 2419 citations between 1999 and 2018, more than 100 of which have been documented since 2012. The highest average citation (citations/article) of ≥10 was observed only in 2006 (mean, 11.00) and 2014 (mean, 10.20).

**Table 1.** Publication trend of articles published by Indian physiotherapists between 1999 and 2018. Data obtained from Web of Science. Includes all articles types. N articles = 488.

Publication year	Articles	% of total publications	Citations total	Average citation (citations/article)
1999	1	0.20	0	0.00
2000	1	0.20	0	0.00
2001	4	0.82	1	0.25
2002	1	0.20	2	2.00
2003	1	0.20	3	3.00
2004	6	1.23	8	1.33
2005	7	1.43	8	1.14
2006	1	0.20	11	11.00
2007	7	1.43	18	2.57
2008	9	1.84	17	1.89
2009	16	3.28	34	2.13
2010	11	2.25	69	6.27
2011	18	3.69	93	5.17
2012	21	4.30	131	6.24
2013	26	5.33	184	7.08
2014	25	5.12	255	10.20
2015	82	16.80	297	3.62
2016	103	21.11	356	3.46
2017	74	15.16	425	5.74
2018	74	15.16	507	6.85
Total	488	100.00	2419	

A total of 264 journals had published the 488 retrieved articles. The top 20 journals in which Indian physiotherapists published over the study period are displayed in Table 2. The top 20 journals published 174 articles i.e., 35.66% of total publications (N=488) in the research period. Out of the top 20 ranked journals, 11 were journals based in India, 26.79% of the total publications.

**Table 2.** Top 20 journals where Indian physiotherapists published between 1999 and 2018. Data obtained from Web of Science. Includes all articles types. N articles = 488.

Journals	Country	h-Index	SJR Value	JCR IF	Articles	% of total articles	Total citations	Average citation (citations/article)
International Journal of Physiotherapy	India	*	*	*	35	7.17	6	0.17
Journal of Evolution of Medical and Dental Sciences- JEMDS	India	*	*	*	30	6.15	4	0.13
Journal of Clinical and Diagnostic Research	India	28	0.35	*	26	5.33	40	1.54
Haemophilia	UK	84	1.16	3.59	7	1.43	68	9.71
Indian Journal of Critical Care Medicine	India	25	0.34	*	7	1.43	53	7.57
Indian Journal of Orthopedics	India	24	0.37	0.978	7	1.43	17	2.43
Journal of Orthopaedic Surgery	France	36	0.43	0.957	6	1.23	36	6
Physiotherapy Theory and Practice	England	39	0.54	1.158	6	1.23	25	4.17
Indian Pediatrics	India	46	0.34	1.163	5	1.02	34	6.8
International Journal of Scientific Study	India	*	*	*	5	1.02	17	3.4
Journal of Back and Musculoskeletal Rehabilitation	Netherlands	25	0.53	0.814	5	1.02	0	0
Nitte University Journal of Health Science	India	*	*	*	5	1.02	0	0
Annals of Indian Academy of Neurology	India	22	0.38	0.898	4	0.82	43	10.75
Hong Kong Physiotherapy Journal	Hong Kong	11	0.3	*	4	0.82	35	8.75
International Journal of Oral and Maxillofacial Surgery	Denmark	90	1.09	1.961	4	0.82	29	7.25
Journal of Maxillofacial Oral Surgery	India	*	*	*	4	0.82	13	3.25
Journal of Physical Therapy Science	Japan	23	0.8	0.392	4	0.82	12	3
Leprosy Review	UK	40	0.48	0.541	4	0.82	5	1.25
Annals of Neurosciences	India	14	0.44	*	3	0.61	16	5.33
Bangladesh Journal of Medical Science	Bangladesh	7	0.15	*	3	0.61	0	0

*=Indexed in Emerging Science Citation Index (ESCI) but not indexed in SJR and JCR

The *International Journal of Physiotherapy* published 35 articles, with six citations for these 35 articles, an average citation per article as 0.17. It was the most active journal found in this study and contributed to 7.17% of total publications. In contrast, the journal *Haemophilia* published seven articles with 68 citations for these articles, an average citation of 9.71. Similarly, *Annals of Indian Academy of Neurology* published four articles, with citations of 43, giving it the highest average citation of 10.75 ( Table 2).

Table 3 shows the top 20 authors who worked with Indian physiotherapists to publish physiotherapy articles. These authors contributed 22.13% of total publications (N=488) in collaboration with Indian physiotherapists. Kumar S, Mahadevappa M, and Samuel AJ collectively have accounted for 5.74% of total publications (N=488). An Indian author named Kumar R (ICMR-National Institute of Occupational Health) is the Indian physiotherapist with the highest h-index (18) and had published five articles, which were cited 1372 times. The citations per article of that particular author was observed as high of 274.40. Further, the type of author collaboration was explored and the year-wise collaboration pattern of the articles was presented in Table 4. Out of 488 publications, 27.05% of articles were published by five and above authors, and 5.5% by a single author.

**Table 3.** Top 20 authors collaborated between 1999 and 2018. Data obtained from Web of Science. Includes all articles types. N articles = 488.

Author	Institution	Country	Articles	%	Total citation	Citation per article	h-index
Kumar S	King George Med University	India	12	2.46	21	1.75	2
Mahadevappa M	JSS Mahavidyapeetha	India	8	1.64	1026	128.25	13
Samuel AJ	Maharishi Markandeshwar	India	8	1.64	20	2.50	2
Biswas A	Jadavpur University	India	6	1.23	544	90.67	15
Singh S	Banaras Hindu University	India	6	1.23	231	38.50	2
Kumar R	ICMR-National Institute of Occupational Health	India	5	1.02	1372	274.40	18
Prakash V	Charotar University of Science and Technology	India	5	1.02	480	96.00	9
Iqbal ZA	King Saud University	Saudi Arabia	5	1.02	119	23.80	6
Lenka PK	National Institute of Occupational Health	India	5	1.02	105	21.00	5
Pattnaik M	National Institute of Technology	India	5	1.02	92	18.40	6
Hariohm K	Center for Evidence based Neurorehabilitation	India	5	1.02	28	5.60	3
Kumar A	Basaveshwara Teaching and General Hospital	India	5	1.02	0	0.00	0
Kumar N	Central Scientific Instruments Organisation	India	5	1.02	0	0.00	0
Gupta A	National Institute of Mental Health and Neurosciences	India	4	0.82	513	128.25	14
Maiya AG	Manipal University	India	4	0.82	53	13.25	7
Goregaonkar AB	Lokmanya Tilak Municipal General Hospital	India	4	0.82	38	9.50	4
Arumugam N	Punjabi University	India	4	0.82	35	8.75	3
Dutta A	North Bengal Medical College	India	4	0.82	33	8.25	3
Gupta M	Vardhaman College of Engineering	India	4	0.82	30	7.50	3
Gupta P	Pt JNM Medical College Raipur	India	4	0.82	2	0.50	2

**Table 4.** Collaboration Patterns of Articles between 1999 and 2018. Data obtained from Web of Science. Includes all articles types. N articles = 488.

Publication year	Single Author	Two Authors	Three Authors	Four Authors	Five and Above Authors	Total Authors
1999			1		0	1
2000				1	0	1
2001	1			2	1	4
2002					1	1
2003	1				0	1
2004	1			2	3	6
2005	3		2	2	0	7
2006				1	0	1
2007			3	1	3	7
2008		3	3	1	2	9
2009		2	5	3	6	16
2010		3	1	5	2	11
2011		3	1	6	8	18
2012	2	2	6	4	7	21
2013	2	5	9	6	4	26
2014		5	3	11	6	25
2015	4	16	19	25	18	82
2016	5	18	32	23	25	103
2017	2	15	16	15	26	74
2018	6	19	16	13	20	74
Grand Total	27	91	117	121	132	488

The top 20 institutions collaborating with Indian physiotherapists for physiotherapy research are displayed in Table 5. Among these institutions, Manipal University (India) has the highest number of publications, with 7.38% of total publications, followed by Christian Medical College Hospital (India; 3.89%), the Indian Institute of Technology (India; 3.69%) and King Saud University (KSU; Saudi Arabia; 3.69%). In total, 90% of collaborating institutions were based in India. Internationally, KSU and the University of London (UK; 1.23%) had the most active cooperation with Indian physiotherapists over this time period.

**Table 5.** Top 20 institutions collaborating with Indian physiotherapists between 1999 and 2018. Data obtained from Web of Science. Includes all articles types. N articles = 488.

Institutions	Country	Articles	% of total articles	Collaboration
Manipal University	India	36	7.38	National
Christian Medical College Hospital	India	19	3.89	National
Indian Institute of Technology	India	18	3.69	National
King Saud University	Saudi Arabia	18	3.69	International
All India Institute of Medical Sciences	India	14	2.87	National
Dr Dy Patil Vidyapeeth Pune	India	12	2.46	National
Nitte Deemed to Be University	India	12	2.46	National
Maharishi Markandeshwar University	India	11	2.25	National
Sri Ramachandra University	India	11	2.25	National
National Institute of Mental Health Neurosciences India	India	10	2.05	National
Indian Institute of Technology IIT Kharagpur	India	8	1.64	National
Apollo Hospital	India	6	1.23	National
Banaras Hindu University	India	6	1.23	National
Charotar University of Science Technology Charusat	India	6	1.23	National
Jamia Millia Islamia	India	6	1.23	National
Punjabi University	India	6	1.23	National
University of London	UK	6	1.23	International
Pgimer Chandigarh	India	5	1.02	National
St John S Medical College	India	5	1.02	National
St John S National Academy of Health Sciences	India	5	1.02	National

Out of the total publications (N=488), articles published by Indian physiotherapists in collaboration with authors belonging to international countries was as follows: United States (4.92%), Saudi Arabia (4.51%), UK (3.69%), Canada (1.84%), and Sweden (1.02%). Italy, Pakistan, Brazil, Australia, Malaysia, and Mexico contributed 0.82% each to total publications ( Table 6). Out of the top 20 countries, Indian physiotherapists collaborated the most with the US (after India), publishing 24 articles, which secured 370 citations (average citation 15.42). Notably, articles published by Indian physiotherapists in collaboration with German authors had the highest number of average citation (41.00), though only three articles were published. Besides, the top 10 highly cited papers during the study period were provided in Table 7. Among those papers, an article published by Singh *et al.* (2008) in the Digest journal of Nanomaterials and Biostructures received 236 citations until 2018 with the average citation of 18.15 per year.

**Table 6.** Top 20 countries collaborating with Indian physiotherapists between 1999 and 2018. Data obtained from Web of Science. Includes all articles types. N articles = 488.

Countries	Articles	% of total articles	Citations	Average citation (Citations/article)
India	488	100	2819	5.78
USA	24	4.92	370	15.42
Saudi Arabia	22	4.51	90	4.09
UK	18	3.69	245	13.61
Canada	9	1.84	29	3.22
Sweden	5	1.02	35	7.00
Italy	4	0.82	52	13.00
Pakistan	4	0.82	50	12.50
Brazil	4	0.82	37	9.25
Australia	4	0.82	23	5.75
Malaysia	4	0.82	11	2.75
Mexico	4	0.82	0	0.00
Germany	3	0.61	123	41.00
Singapore	3	0.61	102	34.00
France	3	0.61	32	10.67
Denmark	3	0.61	14	4.67
Iran	2	0.41	33	16.50
Argentina	2	0.41	12	6.00
Japan	2	0.41	8	4.00
Ethiopia	2	0.41	1	0.50

**Table 7.** Top 10 highly cited papers by Indian physiotherapists between 1999 and 2018. Data obtained from Web of Science. Includes all articles types. N articles = 488.

Article title	Author (Year of Publication)	Journal Title	Total citations	Average citation per year
Nanotechnology in medicine and antibacterial effect of silver nanoparticles	Singh *et al*. (2008)	Digest journal of Nanomaterials and Biostructures	236	18.15
Resting state changes in functional connectivity correlate with movement recovery for BCI and robot-assisted upper-extremity training after stroke	Varkuti *et al*. (2013)	Neurorehabilitation and Neural Repair	108	13.5
An adaptive wearable parallel robot for the treatment of ankle injuries	Jamwal *et al*. (2014)	Ieee-Asme Transactions on Mechatronics	94	13.43
Post-stroke balance training: Role of force platform with visual feedback technique	Srivastava *et al*. (2009)	Journal of the Neurological Sciences	82	6.83
Seroma formation after breast cancer surgery: What we have learned in the last two decades	Srivastava *et al*. (2012)	Journal of Breast Cancer	73	8.11
Comparison of continuous thoracic epidural and paravertebral blocks for postoperative analgesia after minimally invasive direct coronary artery bypass surgery	Dhole *et al*. (2001)	Journal of Cardiothoracic and Vascular Anesthesia	69	3.45
A comprehensive yoga programs improves pain, anxiety and depression in chronic low back pain patients more than exercise: An RCT	Tekur *et al*. (2012)	Complementary Therapies in Medicine	53	5.89
Functional electrical stimulation of dorsiflexor muscle: Effects on dorsiflexor strength, plantarflexor spasticity, and motor recovery in stroke patients	Sabut *et al*. (2011)	Neurorehabilitation	49	4.9
Mounier-Kuhn syndrome: Report of 8 cases of tracheobronchomegaly with associated complications	Menon *et al*. (2008)	Southern Medical Journal	43	3.31
Treatment-induced plasticity in Cerebral Palsy: A diffusion tensor imaging study	Trivedi *et al*. (2008)	Pediatric Neurology	42	3.23

**Figure 4.**
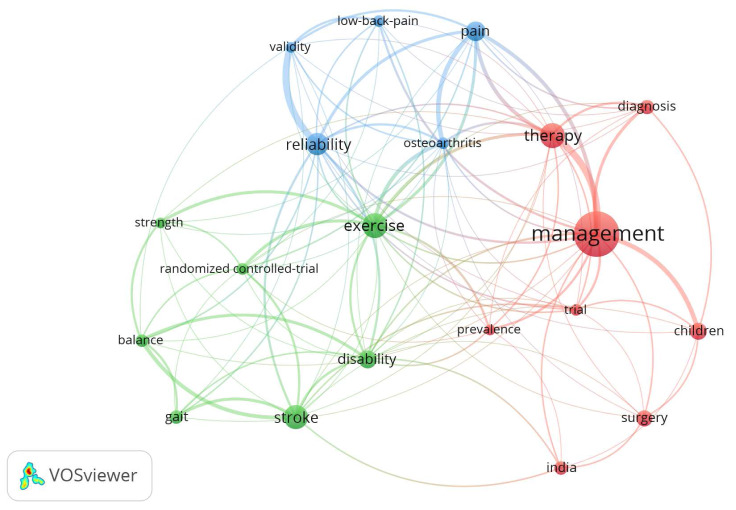
Top 20 keywords co-occurring in articles published by Indian physiotherapists between 1999 and 2018. Graphic created using VOSviewer.

**Figure 5.**
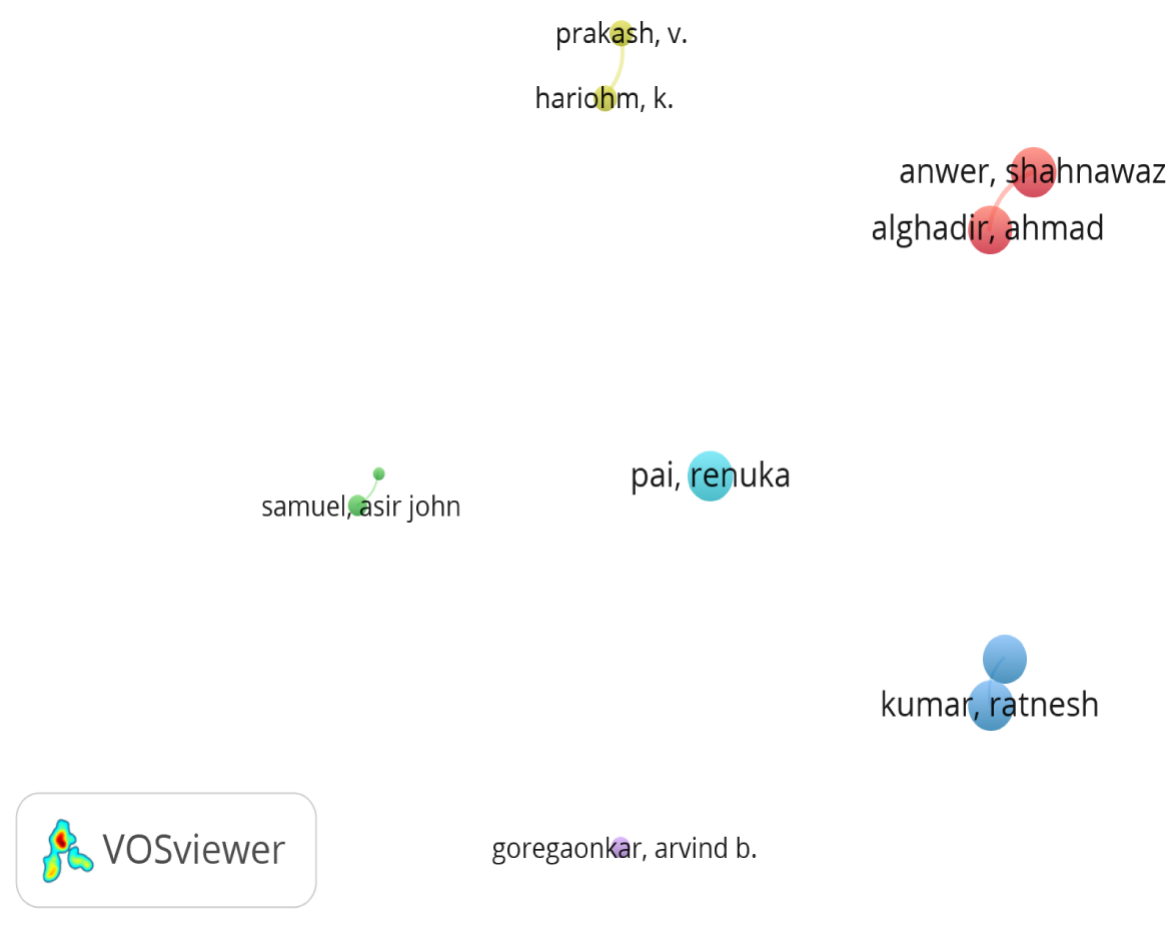
Visualization mapping of top 10 author collaborations.

**Figure 6.**
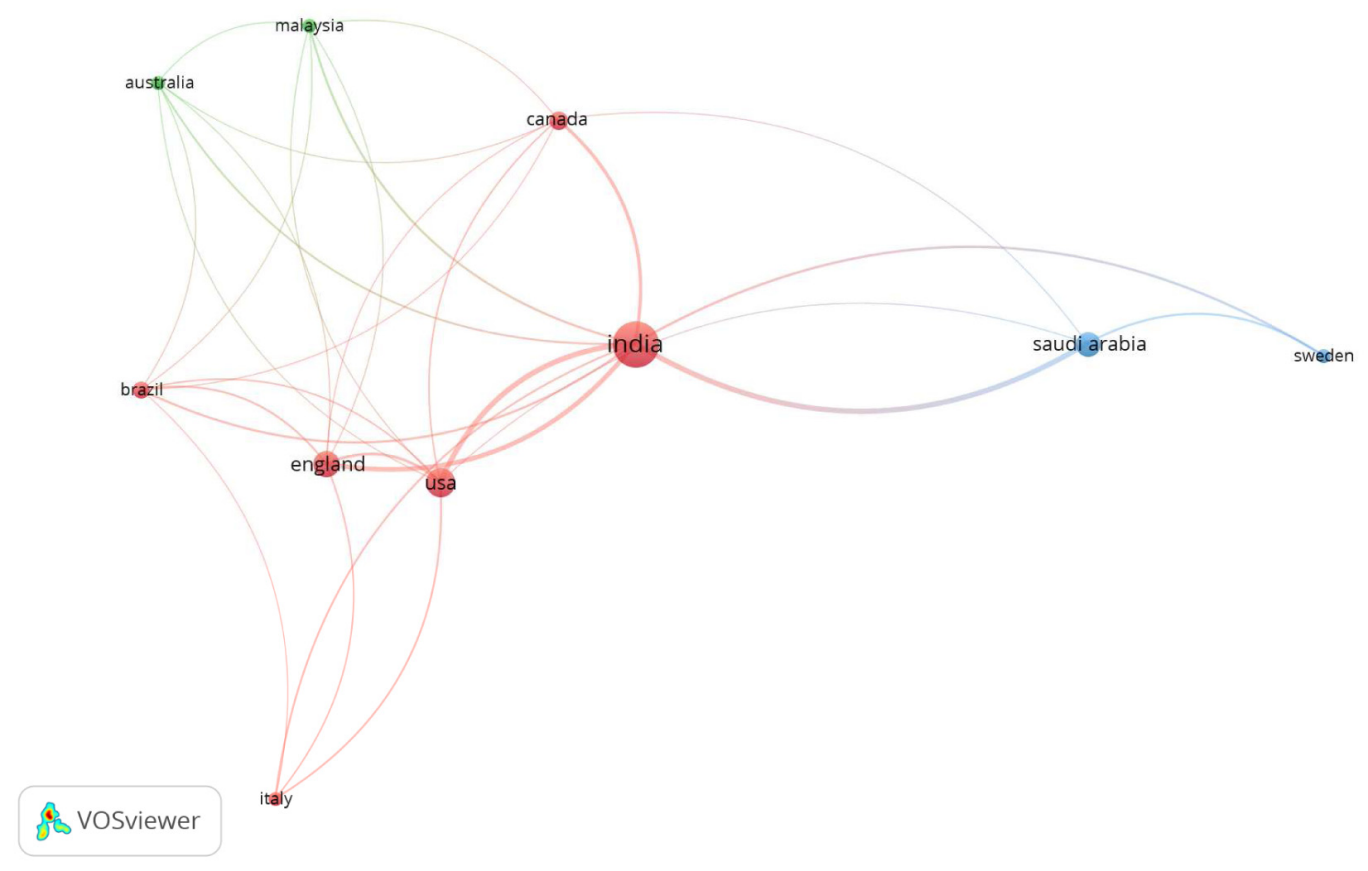
Visualization mapping of top 10 collaborating countries.

Using VOSviewer, the top 20 keywords used in articles (from a total of 2477 keywords) are shown in Figure 4. An article’s keyword may represent its primary material, and to some degree, the frequency of occurrence ^18^. Likewise, co-occurrence can indicate centered themes of research in a field. Through VOSviewer, the authors observed top 20 keywords and it is shown in Table 8. Among the top 20 keywords, the minimum number of occurrences of each keyword was set to 11 and excluded the keyword “Physiotherapy,” “Rehabilitation,” and “Physical Therapy” from the formation of the cluster. There were three co-citation clusters formed using this criterion. The results showed that the keyword “Management (cluster 1 red color)” had the highest linkages (N=50) with all the 3 clusters, followed by keywords “Exercise (cluster 2 green color)” and “Reliability (cluster 3 blue color)” had 40 and 39 linkages respectively with all 3 clusters. Besides, the collaboration observed among the top 10 authors and top 10 countries were presented in Figure 5 and Figure 6 respectively. Concerning the top 10 authors collaboration, co-authorship network analysis produced a map for authors with at least four papers and formed six clusters. The most profile authors in terms of citation were observed as Kumar R and Mahadevappa M. These authors showed more collaboration. Furthermore, network visualization of countries with a minimum of four papers showed the top 10 countries in three clusters. The following pairs of countries showed a strong collaboration between them: India-USA (link strength =22), India-Saudi Arabia (link strength=22), and India-England (link strength=17).

**Table 8.** The top 20 Keywords observed using VOSviewer. Data obtained from Web of Science. Includes all articles types. N articles = 488.

Label	Cluster	Weight<Links>	Weight<Total link strength>	Weight<Occurrences>	Score<Avg. citations>	Score<Avg. norm. citations>
management	1	17	50	46	5.6957	1.1319
exercise	2	16	40	25	4.36	1.2534
reliability	3	14	39	23	6.5652	1.132
therapy	1	14	36	25	9.8	1.7033
pain	3	11	34	20	3.75	1.1042
disability	2	16	30	18	13.6667	1.5366
stroke	2	13	28	24	7.875	1.0259
osteoarthritis	3	12	27	12	6.9167	1.7262
validity	3	11	24	11	2.3636	0.6107
balance	2	9	22	13	11.3846	2.0156
randomized controlled-trial	2	11	19	12	16.5	2.4907
trial	1	11	18	12	7.25	0.6662
diagnosis	1	9	16	15	8.4667	1.7966
children	1	7	15	17	11.0588	0.9079
low-back-pain	3	8	15	12	2.9167	1.7737
gait	2	7	14	14	7.2857	1.2058
prevalence	1	8	14	11	3.7273	0.7486
strength	2	8	14	11	2.6364	0.6387
surgery	1	8	11	16	4.375	0.6347
India	1	6	10	14	6.8571	1.0477

## Discussion

### Publication count

Using the Scopus database, a recent study had observed that Italian physiotherapists published 1083 articles from the year 1995 to 2016. More than 50% of the total publications were produced between the years 2012 and 2016 ^13^. In India, Hariohm *et al.* observed that a considerable increase in the research output of Indian physiotherapists, using the MEDLINE database, between the years 2000 and 2014, with a total of 182 articles ^11^. Through this study, the authors observed that Indian physiotherapists had published 488 articles in WoS from 1999 to 2018, with a peak of 103 articles in 2016. In addition, there was a considerable drop in publication count following 2016. Remarkably, the publication count during the fourth strata (i.e., from 2014 to 2018) accounted for 73.6% (n=358) of total publications (N=488). From these results, it is inferred that Indian physiotherapists are increasingly aware of publishing more articles in high-quality journals in recent years and have enhanced their research competencies gradually to raise their scientific output. Nevertheless, a considerable drop in their publication count after 2016 indicates that there is a need for further research to reveal individual and institutional factors causing this decline and frame appropriate strategies to improve the scientific output of Indian physiotherapists.

### Citations

Littman *et al.* analyzed the research output of 45 physical therapy faculty in the southeastern US from 2000 to 2016 using their curriculum vitae. The range of publications and the citations of these faculty was observed as 0 to 43, and 0 to 943, respectively ^12^. Further, Italian physiotherapists published 1083 articles with 13,373 citations in the Scopus database from before 1995 to 2016 ^13^. Compared to these findings, this study revealed that 488 articles published by Indian physiotherapists in WoS from 1999 to 2018 secured only 2419 citations. Specifically, an article by Singh *et al.* published in 2008 had a high citation count of 236 till 2018. Besides, Sturmer *et al.* found that 222 articles were published by Brazilian physical therapy researchers in WoS in 2010, which had a total of 1805 citations ^6^. In contrast, this study reported that Indian physiotherapists published only 65 articles with 171 citations up to the year 2010 in WoS. Even though the articles published by Indian physiotherapists were suitable enough for several researchers to cite them often, there is a need to improve the citation count of their publications in the future.

### Journals

Notably, this study observed that Indian-based journals published 26.84% of the total publications (N=488); no publications were observed in US-based journals. Further, the highest count of publications was observed in an Indian-based journal *International Journal of Physiotherapy*. This affinity of Indian physiotherapy researchers towards Indian-based journals might be due to the nature of their research articles, or interest in country-based journals. However, those researchers should expand their contribution to other high-quality international journals. Exploring the reasons behind Indian physiotherapists’ choice to publish in these Indian journals is beyond the scope of this study, and further research is warranted to address this critical issue. In general, the choice of researchers to publish in a journal depending on the prestige, impact factor, quality of peer reviews, acceptance rate, readership, article publishing charges, and reputation to the scientific community ^19, 20^. Besides, the journals, such as *Haemophilia* and *Annals of Indian Academy of Neurology*, showed considerable citations and a high average citation for only a few articles published in these journals. This implies that these articles might be more useful for the researchers to cite them often ^21^.

### Collaborating authors

A previous study by Man *et al.* found that four Hong Kong physiotherapy professors had a median h-index of 30.5 and their average total number of citations was 2930.3 ^14^. Moreover, Brazilian physical therapy researchers had a median h-index of 3, according to WoS ^6^. Recently, Vercelli *et al.* reported that the mean h-index of 363 Italian physiotherapists was 2.2, which ranged from 0 to 16; mean citations per author were observed as 58 ^13^. On the other hand, this study observed the top 20 authors who worked with Indian physiotherapists with the range of total citations from 0 to 1372 and h-index from 0 to 18. Particularly, Kumar R (India), had the highest h-index of 18, total citations of 1372, and citations per article of 274.40.

### Collaborating institutions

Hariohm *et al.* revealed that Manipal University is an active research institution with 59 articles in the MEDLINE database from 2000 to 2014 ^11^. In line with this finding, this study also observed that Manipal University in India was the leading one among the top 20 collaborating institutions that had contributed to 7.38% of total publications (N=488). Besides, 90% of these top 20 collaborating institutions were based in India, whereas only two institutions were based in the UK and Saudi Arabia. This implies that Indian physiotherapists had more collaborations with institutions in their own country. However, there is a need for Indian physiotherapists to collaborate with international institutions to improve their scientific output.

### Collaborating countries

This study reveals that Indian physiotherapists published the highest number of articles in collaboration with authors from the following countries, such as the US (4.92% of total publications i.e., N=488) and Saudi Arabia (4.51%). Whereas, the total percentage of publications with other countries is minimal. Hence, this study recommends that Indian physiotherapists should enhance their research collaboration with other countries since collaborative research allows the development of networks with early-career researchers in other countries ^22– 24^, and improves the quality of their scientific output ^25^. Furthermore, earlier studies have also stressed the importance of international research collaboration in health care, and it is frequently regarded as an indicator of quality to develop and disseminate scientific knowledge to newly developing countries ^26, 27^.

### Keywords by co-occurrence

Dash *et al.* stated that the keywords are one of the three pillars of a biomedical research article. Using the right keywords would augment the article being found by other researchers as these are used by abstracting and indexing services ^28^. Hence, this study revealed the top 20 keywords that occurred in various articles using VOSviewer software. It is observed that the keyword “Management” had the highest of 50 linkages with all the three co-citation clusters. 

## Conclusion

This study observed that the scientific output of Indian physiotherapists shows an uptrend in performance since 1999, excluding 2017 and 2018, where a considerable decline was noticed. The results showed that Indian physiotherapists had mostly published in Indian-based journals, and collaborated with Indian institutions. Even though there are high-quality publications, there is a need to enhance both the quality and quantity of scientific papers to increase the high number of citations and average citations. This study also recommends that Indian physiotherapists should expand their research collaboration internationally to improve their scientific output.

### Limitations and recommendations

The findings of this study are only limited to the WoS database. Future research can focus on studying the research output of the Indian physiotherapists in other databases to ascertain their research productivity. Future studies can also focus on analyzing individual and institutional factors influencing the research productivity of Indian physiotherapists and develop suitable strategies to enhance their scientific production.

## Data availability

### Underlying data

Open Science Framework: Visualization pattern of the highly cited scientific output of Indian Physiotherapists: A bibliometric study, https://doi.org/10.17605/OSF.IO/8GSDH ^29^

This project contains the following underlying data:

- Article level and citation data for all 488 articles retrieved.
- Journal, author, institution and country data for all 488 articles retrieved.

Data are available under the terms of the Creative Commons Zero "No rights reserved" data waiver (CC0 1.0 Public domain dedication).

## References

[1] Ilott I Bury T: Research capacity. *Physiotherapy.* 2002;88(4):194–200. 10.1016/S0031-9406(05)60410-5

[2] Ilott I: Challenges and strategic solutions for a research emergent profession. *Am J Occup Ther.* 2004;58(3):347–352. 10.5014/ajot.58.3.347 15202634

[3] Coury HJCG Vilella I: Profile of the Brazilian physical therapy researcher. *Rev Bras Fisioter.* 2009;13(4):356–363. 10.1590/S1413-35552009005000048

[4] Richter RR Schlomer SL Krieger MM: Journal publication productivity in academic physical therapy programs in the United States and Puerto Rico from 1988 to 2002. *Phys Ther.* 2008;88(3):376–386. 10.2522/ptj.20060266 18096652

[5] Mugnaini R Packer AL Meneghini R: Comparison of scientists of the Brazilian Academy of Sciences and of the National Academy of Sciences of the USA on the basis of the *h*-index. *Braz J Med Biol Res.* 2008;41(4):258–262. 10.1590/s0100-879x2008000400001 18392447

[6] Sturmer G Viero CC Silveira MN: Profile and scientific output analysis of physical therapy researchers with research productivity fellowship from the Brazilian National Council for Scientific and Technological Development. *Braz J Phys Ther.* 2013;17(1):41–48. 10.1590/s1413-35552012005000068 23538457

[7] Robertson VJ: Research and the cumulation of knowledge in *Physical Therapy*. *Phys Ther.* 1995;75(3):223–332. 10.1093/ptj/75.3.223 7870753

[8] Frantz JM Rhoda A Struthers P: Research productivity of academics in a physiotherapy department: a case study. *Afr J Health Prof Educ.* 2010;2(2):17–20. Reference Source

[9] Coronado RA Riddle DL Wurtzel WA: Bibliometric analysis of articles published from 1980 to 2009 in *Physical Therapy*, journal of the American Physical Therapy Association. *Phys Ther.* 2011;91(5):642–655. 10.2522/ptj.20100267 21372202

[10] Hariohm K Prakash V Saravankumar J: Quantity and quality of randomized controlled trials published by Indian physiotherapists. *Perspect Clin Res.* 2015;6(2):91–97. 10.4103/2229-3485.154007 25878954 PMC4394587

[11] Hariohm K Prakash V Saravankumar J: Research productivity of Indian physiotherapists: A review of MEDLINE. *Curr Sci.* 2016;110(12):2226–2230. 10.18520/cs/v110/i12/2226-2230

[12] Littman MA Sonne JW Smith GV: Research productivity of doctor of physical therapy faculty promoted in the southeastern United States. *Med Educ Online.* 2017;22(1):1368849. 10.1080/10872981.2017.1368849 28835200 PMC5653937

[13] Vercelli S Ravizzotti E Paci M: Are they publishing? A descriptive cross-sectional profile and bibliometric analysis of the journal publication productivity of Italian physiotherapists. *Arch Physiother.* 2018;8:1. 10.1186/s40945-017-0042-8 29340208 PMC5759900

[14] Man DWK Tsang WSF Lu EY: Bibliometric study of research productivity in occupational therapy and physical therapy/physiotherapy in four Western countries and five Asian countries/regions. *Aust Occup Ther J.* 2019;66(6):690–699. 10.1111/1440-1630.12608 31595529

[15] Li K Rollins J Yan E: Web of Science use in published research and review papers 1997–2017: A selective, dynamic, cross-domain, content-based analysis. *Scientometrics.* 2018;115:1–20. 10.1007/s11192-017-2622-5 29527070 PMC5838136

[16] Van Eck NJ Waltman L Van den Berg J: Visualizing the computational intelligence field. *IEEE Comput Intell M.* 2006;1(4):6–10. 10.1109/MCI.2006.329702

[17] Van Eck NJ Waltman L: VOS: A new method for visualizing similarities between objects.In: Lenz H-J, Decker, R editors. *Advances in data analysis: Studies in Classification, Data Analysis, and Knowledge Organization* Springer: Berlin, Heidelberg;2007;299–306. 10.1007/978-3-540-70981-7_34

[18] Memon AR Vandelanotte C Olds T: Research Combining Physical Activity and Sleep: A Bibliometric Analysis. *Percept Mot Skills.* 2020;127(1):154–181. 10.1177/0031512519889780 31766944

[19] Pepermans G Rousseau S: The decision to submit to a journal: Another example of a valence‐consistent shift? *J Assoc Inf Sci Tech.* 2016;67(6):1372–1383. 10.1002/asi.23491

[20] Mukherjee D: Choosing the Right Journal-A Comprehensive Guide for Early-career Researchers.2018; [Accessed April 20, 2020]. Reference Source

[21] Aksnes DW Langfeldt L Wouters P: Citations, citation indicators, and research quality: An Overview of basic concepts and theories. *SAGE Open.* 2019 10.1177/2158244019829575

[22] Camargo AA Simpson AJ: Collaborative research networks work. *J Clin Invest.* 2003;112(4):468–471. 10.1172/JCI19520 12925684 PMC171397

[23] Carroll JK Albada A Farahani M: Enhancing International Collaboration Among Early Career Researchers. *Patient Educ Couns.* 2010;80(3):417–420. 10.1016/j.pec.2010.06.020 20663630 PMC2930780

[24] Zutshi A McDonald G Kalejs L: Challenges in collaborative writing: Addressing authorship attribution. *Eur Bus Rev.* 2012;24(1):28–46. 10.1108/09555341211191535

[25] Dakik HA Kaidbey H Sabra R: Research productivity of the medical faculty at the American University of Beirut. *Postgrad Med J.* 2006;82(969):462–464. 10.1136/pgmj.2005.042713 16822923 PMC2563770

[26] Kim KW: Measuring international research collaboration of peripheral countries: taking the context into consideration. *Scientometrics.* 2006;66(2006):231–240. 10.1007/s11192-006-0017-0

[27] Freshwater D Sherwood G Drury V: International research collaboration: Issues, benefits and challenges of the global network. *J Res Nurs.* 2007;11(4):295–303. 10.1177/1744987106066304

[28] Dash M: Three pillars of a biomedical research article: The title, Abstract and keywords. *J Health Spec.* 2016;4(3):186–189. 10.4103/2468-6360.186488

[29] Subbarayalu AV Peter M Idhris M: Visualization pattern of the highly cited scientific output of Indian Physiotherapists: A bibliometric study.2020 10.17605/OSF.IO/8GSDH PMC7308915 32595952

