# Supplementary material for: Visualizing highly cited scientific output of Indian physiotherapists: A bibliometric study
Source: F1000Res. 2020 May 19;9:207. Originally published 2020 Mar 24. [Version 2] doi: 10.12688/f1000research.22390.2 (PMC7308915; doi:10.12688/f1000research.22390.2)
Supplement: Peer review history

# Peer review: Reviewer response for version 2

**DOI:** 10.5256/f1000research.26175.r61695

**Published:** 2020-06-17

**Authors:** Prakash Vaidhiyalingam

**Affiliations:** 1 Ashok and Rita Patel Institute of Physiotherapy, Charotar University of Science and Technology, Changa, Gujarat, India

Its a well conceived study exploring key metrics of research work published by Indian Physiotherapists. I think citation-metrics data provided by the authors can be very useful to understand and analyse the publication trend and research productivity of Indian Physiotherapists.

 Key issues:

1. I find the title and the aim is somewhat misleading. The study provided an in-depth citation analysis of all research work published by Indian PTs in the WoS indexed journals not just 'highly cited' articles. An additional/sub analysis highlighted top 20 publications.
2. The authors's attempt at reviewing High-quality research indexed in WoS is justifiable. I found two major issues related to how high quality is defined in this study and how it is interpreted:
  1. High-quality journal or publication is not clearly defined. I suggest a brief discussion on using citation metrics and indexing databases as  a criteria for research quality to be included in Introduction
  2. Inclusion of journals in Emerging Sources Citation Index (ESCI) need clarification. Journals indexed in ESCI is monitored for quality by WoS but unlike the journals indexed in SCIE it doesn't have impact factor. My suggestion is authors may consider adding a brief note on discussing potential confounding effect of adding multiple journal indexes of SCI. I also think the acronyms (SCI-EXPANDED, SSCI, A&HCI, CPCI-S, CPCI-SSH, ESCI, CCR-EXPANDED, IC) need to  be expanded and a brief description of each would help reader to understand similarities and differences among them.
3. How physiotherapy was defined? It is possible that Indian Physiotherapists might have been a part of research teams of Non-Physiotherapy related research. Did the authors screened only articles focused on areas within the scope of physiotherapy. Among Top 10 cited article list, I find the scope of area of articles articles ranked 1, 5, 6 and 10 are not within Physiotherapy. I checked the article authored by Trivedi *et al *(rank 10). The article was published by authors affiliated with department of radiology. I cannot confirm whether the author Richa Trivedi is a physiotherapist based on the affiliation details given in the article. How did the authors of this article confirmed? I suggest authors to verify other articles too. 
4. Discussion can be more structured with less data and more interpretation of data. 

Is the work clearly and accurately presented and does it cite the current literature?

Yes

If applicable, is the statistical analysis and its interpretation appropriate?

Yes

Are all the source data underlying the results available to ensure full reproducibility?

Yes

Is the study design appropriate and is the work technically sound?

Yes

Are the conclusions drawn adequately supported by the results?

Partly

Are sufficient details of methods and analysis provided to allow replication by others?

Yes

Reviewer Expertise:

Knowledge Translation, outcome measurement and outdoor mobility in stroke

I confirm that I have read this submission and believe that I have an appropriate level of expertise to confirm that it is of an acceptable scientific standard, however I have significant reservations, as outlined above.

# Peer review: Reviewer response for version 1

**DOI:** 10.5256/f1000research.24704.r61693

**Published:** 2020-04-07

**Authors:** Aamir Raoof Memon

**Affiliations:** 1 Institute of Physiotherapy and Rehabilitation Sciences, Peoples University of Medical and Health Sciences for Women, Nawabshah, Pakistan

I enjoyed reading the bibliometric analysis of scientific output of physiotherapists from India. The manuscript is interesting but requires improvement in its content. My suggestions are given below:

**Abstract:**

- *Author 1:* “….determined by producing the most articles” should be revised to “determined by **publishing** the most articles”

**Introduction:**

- *Author 2:* “…….Indian physiotherapists up until 2018 (this study took place in 2019)” please delete “(this study took place in 2019)”
- *Author 3:* “…..this study intended to conduct a bibliometric….” should be revised to “this study was planned to conduct a bibliometric”

**Methods:**

- *Author 4:* “…..the following term” should be revised to “……the following keywords” or “……the following query”.
- *Author 5:* Instead of “physical rehabilitation”, authors could have used “rehabilitation”. Also, authors could use the term “exercise therapy” or “manual therapy” or “manipulative therapy” because not all papers use the terms physical therapy or physiotherapy. Please add space between “AND” and “AD”.
- *Author 6:* Please explain the Web of Science indexes (i.e. SCI-EXPANDED, SSCI, A&HCI, CPCI-S, CPCI-SSH, ESCI, CCR-EXPANDED, IC) in full-form.
- *Author 7:* I think there is a typo here “……to gather information….” and should be revised to “…..to gather information on…”.
- *Author 8:* Inclusion criterion 3 (Fig 1) should be “publications with at least one physiotherapist with institutional/author affiliation from India listed as a co-author”. In addition (related to criterion 1), did the authors include peer-reviewed journal publications or meeting abstracts and other document types?
- *Author 9*: What is the significance of adding “Correction”? The common practice is that this category of papers is EXCLUDED from the analysis.
- *Author 10*: “……the information related to h-index was obtained from the Incites in WoS”: please explain h-index and its use here.
- *Author 11*: Please explain the VOS parameters used and cite their original source.
- *Author 12*: “…..used in this study are proprietary…” should be “…..used in this study is a proprietary….”
- *Author 13*: Statistical analysis should present the bibliometric parameters in detail.

**Results:**

- *Author 14*: “Between 1999 and 2008….” this paragraph should be in the past tense, please revise.
- *Author 15*: “….published 174 articles, 35.58%” should be revised to “published 174 articles i.e. 35.58%”.
- *Author 16*: “…11 were journals based” should be revised to “…..11 journals were based”.
- *Author 17*: “average citation of 0.17” should be revised to “average citation per paper as…”.
- *Author 18*: Fig 2: Please check the heading for Flowchart for VOSviewer analysis – I suspect some errors there.
- *Author 19*: Table 1 (please cross-check with the textual description): I see publications depicting a fluctuating pattern - the only rise is seen after 2010 until 2016. Why don't the authors compare the growth according to the strata of 5 years?
- *Author 20*: Fig 3: It would be good to present the citation trend along with the number of publications - please see these papers. For example, see these papers: https://www.ncbi.nlm.nih.gov/pubmed/31766944; https://doi.org/10.3390/clockssleep2020010. ^1^^,^^2^
- *Author 21*: Table 2-5: I think it would be appropriate to list the top 10 instead of 20 – again, this is a common practice in bibliometric studies. I would also want to see top-10 highly cited papers.
- *Author 22*: Table 2: It would be interesting to discuss the impact factor and SJR ranking ( https://www.scimagojr.com/journalrank.php) of these journals. This will reflect the quality and visibility of these journals. I see that most of these journals are indexed in the ESCI - the lowest level of WoS indexes.
- *Author 23*: “International Journal of Scientific Study”: I could not find this journal in the Master List search ( https://mjl.clarivate.com/search-results). I am not really sure about the authenticity of this journal – it looks rather like a questionable journal to me, so I am not sure how its content came up in the results. Please double-check.
- *Author 24*: “The top 20 institutions ……. displayed in Table 4” – this does not look grammatically correct and needs revision. These could be organized in two tables as national (within India) and international (outside India) collaborations.
- *Author 25*: “An article’s keyword may represent its primary…..” needs citation.
- *Author 26*: It would be better to exclude “Physiotherapy” and “Rehabilitation” from the map because they will certainly be the most used. The readers of this paper would rather be interested to see what other terms had frequent occurrence. The authors should also discuss the map (Fig 4) to reflect upon which keywords tend to occur together. For instance, keywords in green (i.e. management, trial, children, diagnosis, surgery, India) tend to belong to the same cluster and are likely to co-occur together. What was the minimum value set for the co-occurrence of a keyword? For example, see this paper: https://www.ncbi.nlm.nih.gov/pubmed/31766944. ^1^
- *Author 27*: Table 3: the column for h-index should come to the right of total citations. Iqbal ZA does not have Indian affiliation, so these names don’t correspond well with the caption of the table. I believe these could be organized in two tables as national (within India) and international (outside India) collaborations. If this is not possible then the authors are advised to revise the caption of the table to bring more clarity in it.
- *Author 28*: Table 4: Please revisit the caption for clarity. Authors should add a column “collaboration” and categorize it as national and international.
- *Author 29*: Table 5: Six institutions with 4 papers are presented but I don't understand how they are ranked (according to the number of citations or citations/paper). As of now, it appears as if they are listed alphabetically. The common method in bibliometric studies is to rank them based on the number of citations.

**Discussion:**

- *Author 30*: Overall, this section is just the repetition of the results and fails to integrate what is known about the topic and what was found.
- *Author 31*: Concerning these two points: “Exploring the reasons…..critical issue” and “This implies that……other researchers”. These are vague arguments – I believe that this would be due to several factors, including the quality of the journal (indexing, metrics), ease of publication (acceptance rate), focus (audience/readership), APCs and reputation to the scientific community. I think authors have failed to give a balanced view here. Most of these journals appear to be of low-quality. Just a point for your understanding is that the IJPT was once involved in deceptive practices (please see this paper: https://www.ncbi.nlm.nih.gov/pubmed/27924967) ^3^, but it has probably improved its practices and has gotten into ESCI.
- *Author 32*: Collaborating authors: It would be appropriate to discuss the average h-index (and its range) and average citations to the papers by Indian authors.
- *Author 33*: I still have confusion regarding the organization and presentation of findings of this paper in the results and discussion sections. Collaborating authors, institutions, and countries should have been leading authors, institutions and countries. Collaborations between countries, institutions, and authors should better be presented through VOSviewer as done in these papers: https://www.ncbi.nlm.nih.gov/pubmed/31766944; https://doi.org/10.3390/clockssleep2020010. ^1^^,^^2^
- *Author 34*: What is the clinical implication of these keywords? Please discuss.
- *Author 35*: Please add a section about the limitations of this study.

**Conclusions:**

- *Author 36*: The decrease in papers during 2017 and 2018 was not “drastic”, please rephrase this.
- *Author 37*: “…….though there are high-quality publications…” this stands as a mere claim and won’t be meaningful without presenting the top-10 highly cited publications.

**Language & grammar: **

- *Author 38*: I noticed grammatical errors at several places, please revise accordingly.

Is the work clearly and accurately presented and does it cite the current literature?

Yes

If applicable, is the statistical analysis and its interpretation appropriate?

Partly

Are all the source data underlying the results available to ensure full reproducibility?

Yes

Is the study design appropriate and is the work technically sound?

Partly

Are the conclusions drawn adequately supported by the results?

Partly

Are sufficient details of methods and analysis provided to allow replication by others?

Partly

Reviewer Expertise:

Physical activity, rehabilitation, exercise, scientometrics, bibliometric analyses

I confirm that I have read this submission and believe that I have an appropriate level of expertise to confirm that it is of an acceptable scientific standard, however I have significant reservations, as outlined above.

## References

[1] : Research Combining Physical Activity and Sleep: A Bibliometric Analysis. *Percept Mot Skills*.2020;127(1) : 10.1177/0031512519889780 154-181 10.1177/0031512519889780 31766944

[2] : Global Research Output on Sleep Research in Athletes from 1966 to 2019: A Bibliometric Analysis. *Clocks & Sleep*.2020;2(2) : 10.3390/clockssleep2020010 99-119 10.3390/clockssleep2020010 PMC744581133089195

[3] : ResearchGate is no longer reliable: leniency towards ghost journals may decrease its impact on the scientific community. *J Pak Med Assoc*.66(12) :1643-1647 27924967

# Response

**Published:** 2020-05-09

**Authors:** Arun Vijay Subbarayalu

**Affiliations:** Imam Abdulrahman Bin Faisal University, Saudi Arabia

**REVIEWER 2**

**Abstract:**

**Author 1: "….determined by producing the most articles" should be revised to "determined by publishing the most articles"**

The correction has been incorporated in the manuscript (Refer Results section of the Abstract).

Under the Results section of the Abstract, it is written as follows:

Manipal University (n=26) was found to be the most active institution for physiotherapy research in India, as determined by publishing the most articles.

**Introduction:**

***Author 2:*** **"…….Indian physiotherapists up until 2018 (this study took place in 2019)" please delete "(this study took place in 2019)"**

The term "this study took place in 2019" is deleted in the Introduction of the manuscript.

Under Introduction,  it is written as follows:

Hence, there is a need for further research that should involve the WoS database to detect high-quality research publications by Indian physiotherapists until 2018.

***Author 3:*** **"…..this study intended to conduct a bibliometric…." should be revised to "this study was planned to conduct a bibliometric"**

The correction has been incorporated in the text of the manuscript.

Under Introduction, it is updated as follows:

Therefore, this study was planned to conduct a bibliometric study on the scientific output of Indian physiotherapists using WoS during the last two decades (from 1999 to 2018).

**Methods:**

**Author 4: "…..the following term" should be revised to "……the following keywords" or "……the following query".**

The correction has been incorporated in the Abstract and Methods sections of the manuscript.

Under Methods section of the Abstract, following updates has been carried out:

A search was carried out using the following keywords "((TS=(physiotherapy) OR TS=("physical rehabilitation") OR TS=("physical therapy")) AND AD=(India))Indexes=SCI-EXPANDED, SSCI, A&HCI, CPCI-S, CPCI-SSH, ESCI, CCR-EXPANDED, IC Timespan=1999-2018".

Under "Article Selection" of the Methods section

The search was conducted on Oct 14 2019. The term 'Indian physiotherapists' denotes physiotherapy professionals employed in any of the academic or clinical establishments in India in the study period. The search was carried out in WoS using the following keywords "((TS=(physiotherapy) OR TS=("physical rehabilitation") OR TS=("physical therapy")) AND AD=(India))Indexes=SCI-EXPANDED, SSCI, A&HCI, CPCI-S, CPCI-SSH, ESCI, CCR-EXPANDED, IC Timespan=1999–2018". The search started from 1999 since this study aimed to retrieve data from the past two decades.

**Author 5: Instead of "physical rehabilitation", authors could have used "rehabilitation". Also, authors could use the term "exercise therapy" or "manual therapy" or "manipulative therapy" because not all papers use the terms physical therapy or physiotherapy. Please add space between "AND" and "AD".**

The search conducted based on TS=" physiotherapy" TS means Topic

We have initially tried our search using all these alternative keywords, i.e., 'rehabilitation', which brought lots of irrelevant content that was not related to physiotherapy rather related to other medical rehabilitation, after that we have excluded the term "rehabilitation" from our search term.

Likewise, while using the keyword "manual therapy" or "manipulative therapy," as of today, we got 510 articles with very meager differences, and few of them irrelevant to physio records. Even we noticed a few extra articles were also added in WoS using our same previous search.

Next, the space between "AND" and "AD" is added in the Abstract and Methods sections of the manuscript.

Under Methods section of the Abstract, we made the following update:

A search was carried out using the following keywords "((TS=(physiotherapy) OR TS=("physical rehabilitation") OR TS=("physical therapy")) AND AD=(India))Indexes=SCI-EXPANDED, SSCI, A&HCI, CPCI-S, CPCI-SSH, ESCI, CCR-EXPANDED, IC Timespan=1999-2018".

Under "Article Selection" of the Methods section

The search was conducted on Oct 14 2019. The term 'Indian physiotherapists' denotes physiotherapy professionals employed in any of the academic or clinical establishments in India in the study period. The search was carried out in WoS using the following keywords "((TS=(physiotherapy) OR TS=("physical rehabilitation") OR TS=("physical therapy")) AND AD=(India))Indexes=SCI-EXPANDED, SSCI, A&HCI, CPCI-S, CPCI-SSH, ESCI, CCR-EXPANDED, IC Timespan=1999–2018". The search started from 1999 since this study aimed to retrieve data from the past two decades.

**Author 6: Please explain the Web of Science indexes (i.e., SCI-EXPANDED, SSCI, A&HCI, CPCI-S, CPCI-SSH, ESCI, CCR-EXPANDED, IC) in full-form.**

(SCI-EXPANDED)-Science Citation Index Expanded

(SSCI)-Social Sciences Citation Index

(A&HCI)-Arts & Humanities Citation Index

(CPCI-S)-Conference Proceedings Citation Index - Science

(CPCI-SSH)-Conference Proceedings Citation Index - Social Sciences & Humanities

(ESCI)-Emerging Sources Citation Index

(CCR Expanded)-Current Chemical Reactions

(IC)-Index Chemicus

As suggested, we have added these full forms in figure 1, instead of Article Selection paragraph under the Methods section ( *This is done as it is a medium-sized article where we need to fit into the word count provided by the Journal*).

**Author 7: I think there is a typo here "……to gather information…." and should be revised to "…..to gather information on…".**

The correction has been incorporated in the text of the manuscript.

Under Data Analysis of the Methods section

Incites was used to gather information on publication year, authorship ranking, source journal productivity, collaborating institutions, country-wise research collaboration, citations, and collaboration pattern of articles. Also, the information related to h-index was obtained from the Incites in WoS.

**Author 8: Inclusion criterion 3 (Fig 1) should be "publications with at least one physiotherapist with institutional/author affiliation from India listed as a co-author". In addition (related to criterion 1), did the authors include peer-reviewed journal publications or meeting abstracts and other document types?**

The authors have updated the correction in Figure 1. With regard to criterion 1, we have included all types of documents, not restricted to peer-reviewed journal publications.

**Author 9: What is the significance of adding "Correction"? The common practice is that this category of papers is EXCLUDED from the analysis.**

As suggested, one item excluded as it is a correction article, and all the tables & graphs updated accordingly.

**Author 10: "……the information related to h-index was obtained from the Incites in WoS": please explain h-index and its use here.**

We have added the following information concerning h-index in "Data Analysis" of the methods section:

Under Data Analysis of the Methods section, we have updated it as follows:  

In addition, the information related to h-index was obtained from the Incites in WoS. Here, the h-index reflects the productivity of authors based on their publication and citation records. It is useful because it discounts the disproportionate weight of highly cited papers or papers that have not yet been cited.

**Author 11: Please explain the VOS parameters used and cite their original source.**

We have added the following literature source to cite VOC parameter within the manuscript:

Under Data Analysis of the Methods section, we have updated it as follows:   

Retrieved articles were analyzed using Incites in WoS and Visualization of Similarities (VOS) viewer 1.6.11. VOS is a new method used for visualizing similarities between objects ^16,17^.

Under References, both the articles reference are added as follows:  

16. Van Eck NJ, Waltman L, Van den Berg J, Kaymak, U: Visualizing the computational intelligence field. *IEEE Comput. Intell. M. *2006;1(4):6-10.

17. Van Eck NJ, Waltman L: VOS: A new method for visualizing similarities between objects. In Lenz H-J, Decker R, editors. Advances in data analysis: Studies in Classification, Data Analysis, and Knowledge Organization. Springer: Berlin, Heidelberg; 2007. p. 299-306. https://doi.org/10.1007/978-3-540-70981-7_34

**Author 12: "…..used in this study are proprietary…" should be "…..used in this study is a proprietary…."**

The required correction carried out. The word 'is a' has been added in front of the word 'proprietary software'.

Under Data Analysis of the Methods section,  we have updated it as follows:    

Since Incites in WoS used in this study is a proprietary software, the researchers could alternatively use a tab-delimited file downloaded from WoS and use it in VOSviewer.

**Author 13: Statistical analysis should present the bibliometric parameters in detail.**

We have included bibliometric parameters related to publication year, authorship ranking, source journal productivity, collaborating institutions, country-wise research collaboration, citations, and h-index. As suggested by the reviewer, we have added a year-wise collaboration pattern of the articles as an additional parameter (Table 4).

Refer to the following table caption in the text of the manuscript.

**Table 4: Collaboration Patterns of Articles between 1999 and 2018.**

Data obtained from Web of Science. Includes all articles types. N articles = 488.

**Results:**

**Author 14: "Between 1999 and 2008…." this paragraph should be in the past tense, please revise.**

The respective paragraph has been revised and presented in the past tense. The revised paragraph is provided in the Results section as follows:

Under the Results section

The chosen period of study was divided into four strata with five years each. The strata were 1999-2003; 2004-2008; 2009-2013; and 2014-2018. In the first two strata, the publication count was observed in a single digit (≤9). In the third strata, this count has reached two digits with a maximum of 26 in 2013. However, in the fourth strata, an abrupt rise in the publication count was observed with a peak of 103 in 2016. Notably, the publication count after 2016 decreased and rose slightly in 2018, but not to those levels seen in 2016 (Table 1 and Figure 3). Regarding citation count, there was a gradual rise over the research period, with a total of 2419 citations between 1999 and 2018, more than 100 of which have been documented since 2012. The highest average citation (citations/article) of ≥10 was observed only in 2006 (mean, 11.00) and 2014 (mean, 10.20).

**Author 15: "….published 174 articles, 35.58%" should be revised to "published 174 articles i.e. 35.58%".**

The correction has been incorporated in the text of the manuscript.

Under the Results section as follows:

A total of 264 journals had published the 488 retrieved articles. The top 20 journals in which Indian physiotherapists published over the study period are displayed in Table 2. The top 20 journals published 174 articles, i.e., 35.66% of total publications (N=488) in the research period. Out of the top 20 ranked journals, 11 journals were based in India, 26.84% of the total publications.

**Author 16: "…11 were journals based" should be revised to "…..11 journals were based".**

The correction has been incorporated in the text of the manuscript.

Under the Results section as follows:

A total of 264 journals had published the 488 retrieved articles. The top 20 journals in which Indian physiotherapists published over the study period are displayed in Table 2. The top 20 journals published 174 articles i.e., 35.66% of total publications (N=488) in the research period. Out of the top 20 ranked journals, 11 journals were based in India, 26.84% of the total publications.

**Author 17: "average citation of 0.17" should be revised to "average citation per paper as…".**

The correction has been incorporated in the text of the manuscript.

Under the Results section, we have updated it as follows:    

The *International Journal of Physiotherapy* published 35 articles, with six citations for these 35 articles, an average citation per article as 0.17. It was the most active Journal found in this study and contributed to 7.17% of total publications. In contrast, the journal *Haemophilia* published seven articles with 68 citations for these articles, an average citation of 9.71. Similarly, *Annals of Indian Academy of Neurology* published four articles, with citations of 43, giving it the highest average citation of 10.75 ( Table 2).

**Author 18: Fig 2: Please check the heading for Flowchart for VOSviewer analysis – I suspect some errors there.**

In the flow chart, we have mentioned the subject, and, in the graph, we have indicated the keyword.  To address this issue, we have updated the Figure 2 by providing the following corrections on it.

 

- In the heading box “Flowchart….VOSviewer software”, we changed the term "co-occurrence of subjects" to "co-occurrence of keywords."

 

- The heading in the second box of figure 2, we changed the term "co-occurrence of subjects" to "co-occurrence of keywords."

 

In addition, in Step-5 box of Figure 2, the number 489 is replaced with 488.

**Author 19: Table 1 (please cross-check with the textual description): I see publications depicting a fluctuating pattern - the only rise is seen after 2010 until 2016. Why don't the authors compare the growth according to the strata of 5 years?**

As advised, we have added the following description in the results section: 

Under the Results section

The chosen period of study was divided into four strata with five years each. These strata were 1999-2003; 2004-2008; 2009-2013; and 2014-2018. In the first two strata, the publication count was observed in a single digit (≤9). In the third strata, this count has reached two digits with a maximum of 26 in 2013. However, in the fourth strata, an abrupt rise in the publication count was observed with a peak of 103 in 2016. Notably, the publication count after 2016 decreased and rose slightly in 2018, but not to those levels seen in 2016 (Table 1 and Figure 3).

**Author 20: Fig 3: It would be good to present the citation trend along with the number of publications - please see these papers. For example, see these papers: https://www.ncbi.nlm.nih.gov/pubmed/31766944; https://doi.org/10.3390/clockssleep202001 0.1,2**

The corresponding figure 3 is modified as the same provided in the example study. Also, the figure caption is revised as follows: Figure 3. The growth trend for publications and citations by Indian physiotherapists between 1999 and 2018.

**Author 21: Table 2-5: I think it would be appropriate to list the top 10 instead of 20 – again, this is a common practice in bibliometric studies. I would also want to see top-10 highly cited papers.**

The focus of our study is to review the results based on the top 20 journals, authors, institutions, and countries collaborating with Indian physiotherapists. However, as per the reviewer's feedback, we have provided information about the top 10 highly cited papers in Table 7 of the manuscript. The description of the top 10 highly cited articles is provided in the Results and Discussion section.

Under the Results section, we have updated it as follows:    

Besides, the top 10 highly cited papers during the study period were provided in Table 7. Among those papers, an article published by Singh et al. (2008) in the Digest journal of Nanomaterials and Biostructures received 236 citations until 2018 with the average citation of 18.15 per year.

(Refer Table 7)

Under Discussion section, we have updated it as follows:    

Citations

Littman *et al.* analyzed the research output of 45 physical therapy faculty in the southeastern US from 2000 to 2016 using their curriculum vitae. The range of publications and the citations of these faculty was observed as 0 to 43, and 0 to 943, respectively ^12^ . Further, Italian physiotherapists published 1083 articles with 13,373 citations in the Scopus database from before 1995 to 2016. ^13^ Compared to these findings, this study revealed that 488 articles published by Indian physiotherapists in WoS from 1999 to 2018 secured only 2419 citations. Specifically, an article by Singh M published in 2008 had a high citation count of 236 till 2018. Besides, Sturmer *et al.* found that 222 articles were published by Brazilian physical therapy researchers in WoS in 2010, which had a total of 1805 citations ^6^ . In contrast, this study reported that Indian physiotherapists published only 65 articles with 171 citations up to the year 2010 in WoS. Even though the articles published by Indian physiotherapists were suitable enough for several researchers to cite them often, there is a need to improve the citation count of their publications in the future.

**Author 22: Table 2: It would be interesting to discuss the impact factor and SJR ranking (https://www.scimagojr.com/journalrank.php) of these journals. This will reflect the quality and visibility of these journals. I see that most of these journals are indexed in the ESCI - the lowest level of WoS indexes.**

This study is focused on revealing the scientific output of Indian physiotherapists in the WoS database during the period from 1999 to 2018. The criteria have been fixed that this study is limited to the WoS database, and the Web of Science indexes covered was mentioned in the search items (i.e., SCI-EXPANDED, SSCI, A&HCI, CPCI-S, CPCI-SSH, ESCI, CCR-EXPANDED, IC). As advised by the reviewer, the h-index, JCR impact factor and SJR ranking of the observed journals are additionally provided in Table 2 (Refer Table 2.)

**Author 23: "International Journal of Scientific Study": I could not find this Journal in the Master List search (https://mjl.clarivate.com/search-results). I am not really sure about the authenticity of this Journal – it looks rather like a questionable journal to me, so I am not sure how its content came up in the results. Please double-check.**

This Journal is indexed in Web of Science till 2017. Kindly refer to the following screenshot.

**Author 24: "The top 20 institutions ……. displayed in Table 4" – this does not look grammatically correct and needs revision. These could be organized in two tables as national (within India) and international (outside India) collaborations.**

The corresponding sentence is revised for grammatical correction and provided in the Results section.

Under Results, we have updated it as follows:    

The top 20 institutions collaborating with Indian physiotherapists for physiotherapy research are displayed in Table 5.

In addition, the authors have added a column titled "Collaboration" in Table 5 to mention the collaborating institutions as "National" or "International" (Refer Table 5).

**Author 25: "An article's keyword may represent its primary….." needs citation.**

As advised, the reference for the following sentence "An article's keyword may represent its primary….." is provided in the Results section.

Under the Results section

An article's keyword may represent its primary material, and to some degree, the frequency of occurrence ^18^.

**Author 26: It would be better to exclude "Physiotherapy" and "Rehabilitation" from the map because they will certainly be the most used. The readers of this paper would rather be interested to see what other terms had frequent occurrence. The authors should also discuss the map (Fig 4) to reflect upon which keywords tend to occur together. For instance, keywords in green (i.e., management, trial, children, diagnosis, surgery, India) tend to belong to the same cluster and are likely to co-occur together. What was the minimum value set for the co-occurrence of a keyword? For example, see this paper: https://www.ncbi.nlm.nih.gov/pubmed/31766944.1**

The authors have discussed the map (Figure 4) to reflect upon which keywords tend to occur together. They have also described the minimum value set for the co-occurrence of a keyword. The description is provided in the Results section (Also Refer to Figure 4 and Table 8).

Under the Results section, it is updated as follows:

Using VOSviewer, the top 20 keywords used in articles (from a total of 2477 keywords) are shown in Figure 4.  An article's keyword may represent its primary material, and to some degree, the frequency of occurrence ^18^. Likewise, co-occurrence can indicate centered themes of research in a field. Through VOSviewer, the authors observed the top 20 keywords, and it is shown in Table 8. Among the top 20 keywords, the minimum number of occurrences of each keyword was set to 11 and excluded the keyword "Physiotherapy," "Rehabilitation," and "Physical Therapy" from the formation of the cluster. There were three co-citation clusters formed using this criterion. The results showed that the keyword "Management (cluster 1 red color)" had the highest linkages (N=50) with all the 3 clusters, followed by keywords "Exercise (cluster 2 green color)" and "Reliability (cluster 3 blue color)" had 40 and 39 linkages respectively with all 3 clusters.

**Author 27: Table 3: the column for h-index should come to the right of total citations. Iqbal ZA does not have Indian affiliation, so these names don't correspond well with the caption of the table. I believe these could be organized in two tables as national (within India) and international (outside India) collaborations. If this is not possible then the authors are advised to revise the caption of the table to bring more clarity in it.**

As advised, in Table 3, we have relocated the h-index column to the right side of the total citations column. The new column "citations per article" is also added. In addition, Table 3 caption has already been edited as follows:

Table 3. Top 20 Authors collaborated between 1999 and 2018.

**Author 28: Table 4: Please revisit the caption for clarity. Authors should add a column "collaboration" and categorize it as national and international.**

Table 4 is changed into Table 5. We have changed the caption of Table 5 as "Top 20 institutions collaborating with Indian physiotherapists between 1999 and 2018. A new column, "collaboration," has been added to categorize the institutions as national or international collaboration (Refer to Table 5).

**Author 29: Table 5: Six institutions with 4 papers are presented, but I don't understand how they are ranked (according to the number of citations or citations/paper). As of now, it appears as if they are listed alphabetically. The common method in bibliometric studies is to rank them based on the number of citations.**

Table 5 is renamed as Table 6. The order of the collaborating countries is listed on the basis of the number of articles. Further, the countries with similar number of articles are ranked based on the number of citations and it is presented in Table 6.

**Discussion:**

**Author 30: Overall, this section is just the repetition of the results and fails to integrate what is known about the Topic and what was found.**

As advised, the discussion is revised to avoid the repetition of the results and integrate what is know about the Topic and what was found.

**Author 31: Concerning these two points: "Exploring the reasons…..critical issue" and "This implies that……other researchers". These are vague arguments – I believe that this would be due to several factors, including the quality of the journal (indexing, metrics), ease of publication (acceptance rate), focus (audience/readership), APCs and reputation to the scientific community. I think authors have failed to give a balanced view here. Most of these journals appear to be of low-quality. Just a point for your understanding is that the IJPT was once involved in deceptive practices (please see this paper: https://www.ncbi.nlm.nih.gov/pubmed/27924967)3, but it has probably improved its practices and has gotten into ESCI.**

As suggested, we have modified the sentence as follows:

Under the heading "Journals" in the Discussion section, we have updated it as follows:

Exploring the reasons behind Indian physiotherapists' choice to publish in these Indian journals is beyond the scope of this study, and further research is warranted to address this critical issue. In general, the choice of researchers to publish in a journal depending on the prestige, impact factor, quality of peer reviews, acceptance rate, readership, article publishing charges, and reputation to the scientific community ^19,20^.

Under the heading "Journals" in the Discussion section, we have updated it as follows:

As the journals such as Haemophilia and Annals of Indian Academy of Neurology with a considerable citation and a high average citation for only a few articles, the authors mentioned the following sentence:

This implies that these articles might be more useful for the researchers to cite them often ^21^.

**Author 32: Collaborating authors: It would be appropriate to discuss the average h-index (and its range) and average citations to the papers by Indian authors.**

As the average h-index and average citations to the papers by Indian authors were not observed, this study described the range of h-index and total citations of the top 20 authors. A  description is provided under the heading ‘Collaborating authors’ in the discussion section as follows:

**Collaborating authors**

A previous study by Man *et al.* found that four Hong Kong physiotherapy professors had a median h-index of 30.5 and their average total number of citations was 2930.3 ^14^ . Moreover, Brazilian physical therapy researchers had a median h-index of 3, according to WoS ^6^ . Recently, Vercelli *et al.* reported that the mean h-index of 363 Italian physiotherapists was 2.2, which ranged from 0 to 16; mean citations per author were observed as 58 ^13^ . On the other hand, this study observed the top 20 authors who worked with Indian physiotherapists with the range of total citations from 0 to 1372 and h-index from 0 to 18. Particularly, Kumar R (India), had the highest h-index of 18, total citations of 1372, and citations per article of 274.40.

**Author 33: I still have confusion regarding the organization and presentation of findings of this paper in the results and discussion sections. Collaborating authors, institutions, and countries should have been leading authors, institutions and countries. Collaborations between countries, institutions, and authors should better be presented through VOSviewer as done in these papers: https://www.ncbi.nlm.nih.gov/pubmed/31766944; https://doi.org/10.3390/clockssleep202001 0.1,2**

As recommended by the reviewer, the authors have provided information about the top 10 author collaborations and the top 10 collaborating countries in figures (Refer Figure 5 and Figure 6) and cited within the text (Refer Results section).  

Under Results section

Besides, the collaboration observed among the top 10 authors and top 10 countries were presented in figures 5 and 6 respectively. Concerning the top 10 authors collaboration, co-authorship network analysis produced a map for authors with at least four papers and formed six clusters. The most profile authors in terms of citation were observed as Kumar R and Mahadevappa M. These authors showed more collaboration. Furthermore, network visualization of countries with a minimum of four papers showed the top 10 countries in three clusters. The following pairs of countries showed a strong collaboration between them: India-USA (link strength =22), India-Saudi Arabia (link strength=22), and India-England (link strength=17).

**Author 34: What is the clinical implication of these keywords? Please discuss.**

The clinical implications of the keywords were discussed with the appropriated literature under the heading "Keywords by co-occurrence" in the Discussion section.

Refer to the heading "Keywords by co-occurrence" in the Discussion section

Dash *et al.* stated that the keywords are one of the three pillars of a biomedical research article. Using the right keywords would augment the article being found by other researchers as these are used by abstracting and indexing services ^28^.  Hence, this study revealed the top 20 keywords that occurred in various articles using VOSviewer software. It is observed that the keyword "Management" had the highest of 50 linkages with all the three co-citation clusters.

**Author 35: Please add a section about the limitations of this study.**

As advised, a new heading "Limitations and Recommendations" is added in the manuscript.

Limitations and Recommendations

The findings of this study are only limited to the WoS database. Future research can focus on studying the research output of the Indian physiotherapists in other databases to ascertain their research productivity. Future studies can focus on analyzing individual and institutional factors influencing the research productivity of Indian physiotherapists and develop suitable strategies to enhance their scientific production.

**Conclusions:**

**Author 36: The decrease in papers during 2017 and 2018 was not "drastic", please rephrase this.**

The term "drastic" is revised and described as follows in the Conclusion section.

Under Conclusion

**Conclusion**

This study observed that the scientific output of Indian physiotherapists shows an uptrend in performance since 1999, excluding 2017 and 2018, where a considerable decline was noticed. The results showed that Indian physiotherapists had mostly published in Indian-based journals, and collaborated with Indian institutions. Even though there are high-quality publications, there is a need to enhance both the quality and quantity of scientific papers to increase the high number of citations and average citations. This study also recommends that Indian physiotherapists should expand their research collaboration internationally to improve their scientific output.

**Author 37: "…….though there are high-quality publications…" this stands as a mere claim and won't be meaningful without presenting the top-10 highly cited publications.**

As advised, the top 10 highly cited papers are provided in Table 7 of the manuscript. A description of the top 10 highly cited papers is provided in the Results and Discussion section.

Refer to the Results section to see the changes made as follows:

Besides, the top 10 highly cited papers during the study period were provided in Table 7. Among those papers, an article published by Singh et al. (2008) in the Digest journal of Nanomaterials and Biostructures received 236 citations until 2018 with the average citation of 18.15 per year.

Refer to the Discussion section to see the changes made as follows

Citations

Littman *et al.* analyzed the research output of 45 physical therapy faculty in the southeastern US from 2000 to 2016 using their curriculum vitae. The range of publications and the citations of these faculty was observed as 0 to 43, and 0 to 943, respectively ^12^ . Further, Italian physiotherapists published 1083 articles with 13,373 citations in the Scopus database from before 1995 to 2016. ^13^ Compared to these findings, this study revealed that 488 articles published by Indian physiotherapists in WoS from 1999 to 2018 secured only 2419 citations. Specifically, an article by Singh M published in 2008 had a high citation count of 236 till 2018. Besides, Sturmer *et al.* found that 222 articles were published by Brazilian physical therapy researchers in WoS in 2010, which had a total of 1805 citations ^6^ . In contrast, this study reported that Indian physiotherapists published only 65 articles with 171 citations up to the year 2010 in WoS. Even though the articles published by Indian physiotherapists were suitable enough for several researchers to cite them often, there is a need to improve the citation count of their publications in the future.

**References**

Seven new references are added in the revised manuscript.

The newly added references are as follows:

16. Van Eck NJ, Waltman L, Van den Berg J, Kaymak, U: Visualizing the computational intelligence field. *IEEE Comput. Intell. M. *2006;1(4):6-10.

17. Van Eck NJ, Waltman L: VOS: A new method for visualizing similarities between objects. In Lenz H-J, Decker R, editors. Advances in data analysis: Studies in Classification, Data Analysis, and Knowledge Organization. Springer: Berlin, Heidelberg; 2007. p. 299-306. https://doi.org/10.1007/978-3-540-70981-7_34

18. Memon AR., Vandelanotte C, Olds T, Duncan MJ, et al.: Research combining physical activity and sleep: A bibliometric analysis. *Percept. Mot. Skills*, 2020;127(1):154–181. https://doi.org/10.1177/0031512519889780

19. Pepermans G, Rousseau S: The decision to submit to a journal: Another example of a valence‐consistent shift? *J Assoc Inf Sci Tech* *.* 2016;67(6):1372–1383.

20. Mukherjee D: Choosing the Right Journal-A Comprehensive Guide for Early-career Researchers. 2018 Aug 03. https://blog.typeset.io/choose-right-journal-early-stage-researchers-guide-ea2cf236dde4 [Accessed April 20, 2020].

21. Aksnes DW, Langfeldt L, Wouters P: Citations, citation indicators, and research quality: An Overview of basic concepts and theories. *SAGE Open*. 2019 Feb 07. https://doi.org/10.1177/2158244019829575

28. Dash M: Three pillars of a biomedical research article: The title, Abstract and keywords. *J Health Spec.* 2016;4:186-189.

# Peer review: Reviewer response for version 1

**DOI:** 10.5256/f1000research.24704.r61694

**Published:** 2020-04-06

**Authors:** Gopal Nambi

**Affiliations:** 1 Department of Physical Therapy and Health Rehabilitation, College of Applied Medical Sciences, Prince Sattam Bin Abdulaziz University, Al-Kharj, Saudi Arabia

Thank you for the chance to review this paper titled “Visualizing highly cited scientific output of Indian physiotherapists: A bibliometric study”. The major strength of this paper is that they have attempted to undertake a study on this title.

 The title is interesting. The design and outcome measures are appropriate to answer the question and I’m sure a lot of hard work went into conducting this study.

**Abstract:**

- The search is limited with one database (WOS), what about the other databases?

**Introduction:**

- The introduction part is superficial.
- Why is the search duration limited to 1999 – 2018?

**Methods:**

- How many reviews were collected in the articles and what are their kappa score?
- Indian physiotherapist means, who works only in India or Indian physiotherapists works throughout the world?
- Selection criteria should be more specific.

**Discussion:**

- The strength and the limitations of this study are not mentioned.

**Conclusion: **

- Rephrase the conclusion and make it more precise and clear.

Is the work clearly and accurately presented and does it cite the current literature?

Yes

If applicable, is the statistical analysis and its interpretation appropriate?

Not applicable

Are all the source data underlying the results available to ensure full reproducibility?

Partly

Is the study design appropriate and is the work technically sound?

Yes

Are the conclusions drawn adequately supported by the results?

Yes

Are sufficient details of methods and analysis provided to allow replication by others?

Yes

Reviewer Expertise:

Rehabilitation, Sports injuries, Physical Therapy

I confirm that I have read this submission and believe that I have an appropriate level of expertise to confirm that it is of an acceptable scientific standard.

# Response

**Published:** 2020-05-09

**Authors:** Arun Vijay Subbarayalu

**Affiliations:** Imam Abdulrahman Bin Faisal University, Saudi Arabia

**REVIEWER 1**

**Abstract:**

**The search is limited with one database (WOS), what about the other databases?**

As this study only focused on revealing the scientific output of Indian physiotherapists in the WoS database, the authors did not include other databases. However, this statement has been written as a limitation, and a recommendation is suggested. The description is provided under the heading 'Limitations and Recommendations' as follows.

Limitations and Recommendations

The findings of this study are only limited to the WoS database. Future research can focus on studying the research output of the Indian physiotherapists in other databases to ascertain their research productivity. Future studies can focus on analyzing individual and institutional factors influencing the research productivity of Indian physiotherapists and develop suitable strategies to enhance their scientific production.

**Introduction:**

**The introduction part is superficial.**

Since it is a medium article, the introduction part is briefly written to cover the scientific output of physiotherapists and physiotherapy research in the Indian context. 

**Why is the search duration limited to 1999 – 2018?**

In the Indian context, Hariohm et al. have studied the research productivity of the Indian physiotherapists in the Medline database from 2000 to 2014. Hence, this study aimed to reveal the scientific output of Indian physiotherapists in the WoS database for the past two decades, i.e., 1999 to 2018. This study was conducted in the year 2019.

**Methods:**

**How many reviews were collected in the articles, and what are their kappa score?**

This study found 53 reviews by Indian physiotherapists during the period between 1999 and 2018. As this study only focused on revealing the scientific output of Indian physiotherapists in the WoS database, the authors did not explain the Kappa score for analysis and discussion.

**Indian Physiotherapist means, who works only in India or Indian physiotherapists works throughout the world?**

We mean those Indian physiotherapists who work in any of the academic or clinical establishments located in India (refer to article selection paragraph in the manuscript).

It is explained Under "Article Selection" of the Methods section

The search was conducted on Oct 14, 2019. The term 'Indian physiotherapists' denotes physiotherapy professionals employed in any of the academic or clinical establishments in India in the study period. The search was carried out in WoS using the following keywords "((TS=(physiotherapy) OR TS=("physical rehabilitation") OR TS=("physical therapy")) AND AD=(India))Indexes=SCI-EXPANDED, SSCI, A&HCI, CPCI-S, CPCI-SSH, ESCI, CCR-EXPANDED, IC Timespan=1999–2018". The search started from 1999 since this study aimed to retrieve data from the past two decades.

**Selection criteria should be more specific.**

We have included four selection criteria (i) Only those publications made by Indian Physiotherapist who works in India (subject understudy); (ii) Publications made between the year 1999 to 2018 (Timespan) (iii) database explored and the exact time where this search was carried out and (iv) key words used to retrieve the articles. (refer to article selection paragraph & figure 1)

**Discussion:**

**The strength and limitations of this study are not mentioned.**

The strength of this study is observed as the uptrend in the scientific output of Indian physiotherapists since 1999. It is mentioned in the conclusion section. Further, the limitations and recommendations of this study are mentioned under the heading "Limitations and Recommendations".

The following description is added Under 'Conclusion section.'

This study observed that the scientific output of Indian physiotherapists shows an uptrend in performance since 1999, excluding 2017 and 2018, where a considerable decline was noticed. The results showed that Indian physiotherapists had mostly published in Indian-based journals, and collaborated with Indian institutions. Even though there are high-quality publications, there is a need to enhance both the quality and quantity of scientific papers to increase the high number of citations and average citations. This study also recommends that Indian physiotherapists should expand their research collaboration internationally to improve their scientific output.

The following description is added Under 'Limitations and Recommendations' section

The findings of this study are only limited to the WoS database. Future research can focus on studying the research output of the Indian physiotherapists in other databases to ascertain their research productivity. Future studies can focus on analyzing individual and institutional factors influencing the research productivity of Indian physiotherapists and develop suitable strategies to enhance their scientific production.

**Conclusion:**

**Rephrase the Conclusion and make it more precise and clear.**

The Conclusion is revised to improve the clarity of the readers as follows:

Under Conclusion

This study observed that the scientific output of Indian physiotherapists shows an uptrend in performance since 1999, excluding 2017 and 2018, where a considerable decline was noticed. The results showed that Indian physiotherapists had mostly published in Indian-based journals, and collaborated with Indian institutions. Even though there are high-quality publications, there is a need to enhance both the quality and quantity of scientific papers to increase the high number of citations and average citations. This study also recommends that Indian physiotherapists should expand their research collaboration internationally to improve their scientific output.
